# Temperament and Character Domains of Personality and Depression

**DOI:** 10.1155/2011/765691

**Published:** 2012-04-18

**Authors:** Toshinori Kitamura, C. Robert Cloninger

**Affiliations:** ^1^Kitamura Institute of Mental Health Tokyo, 8-12-4-305 Akasaka, Tokyo 107-0052, Japan; ^2^Department of Psychiatry, Washington University in St. Louis, 660 South Euclid Avenue, St. Louis, MO 63130, USA

The link between personality and depression has long intrigued researchers and clinicians alike. Personality has been viewed as contributing to the onset and course of depression as well as influencing therapeutic choices for depression. Two major current personality theories are the “Big Five,” in which the NEO-PI is used as a measuring instrument, and the Psychobiology Theory of Personality, which uses the Temperament and Character Inventory (TCI) as a measuring instrument. This special issue deals with the latter theory in terms of its interrelations with depression and related conditions.

The last couple of decades have witnessed a great number of research reports on this topic. The association of TCI dimension with diverse types of health problems, and depression in particular, has been reported in the literature. The TCI has also been studied in terms of predicting treatment responses of depressed patients. Genetic and environmental correlates of TCI dimensions are a hot topic among researchers. Hence we believe that the present special issue is very timely.

This issue consists of six reports. K. Josefsson and colleagues, in Finland, present results from a longitudinal study of young Finns. Based on TCI scores at Time 1, the group tried to predict levels of depression 10 years later. They found that both high harm avoidance (HA) and low self-directedness (SD) independently predicted later depression severity. Thus, a prospective population-based design yielded findings that echoed the results of past cross-sectional and clinical treatment studies.

In a two-year follow-up study of a clinical population of depression, J. G. Goekoop and colleagues in the Netherland reported that only the increase in SD (in this two-year period) was related to the decrease in emotional dysregulation symptoms, while the increase in SD was associated with the decrease in HA. This suggests that symptomatic recovery follows reversibility of lowered SD.

People with current depression may be diagnosed with bipolar disorder if they have a lifelong history of manic or hypomanic episodes. Hence the association of TCI profiles with depression should be examined in terms of previous diagnoses of mood disorders. J. A. Harley and colleagues, in New Zealand, relate the results of their South Island Bipolar Study, namely, that high HA scores differentiated people with major depressive disorder (MDD) and those with bipolar disorder (BD) from unaffected relatives of bipolar probands after controlling for the current severity of depression. HA, however, failed to differentiate those with MDD from those with BD. On the other hand, high self-transcendence (ST) differentiated people with bipolar I (major depression with manic episodes) from those with MDD and unaffected relatives, confirming other reports of the importance of self-transcendence in the creativity of people with bipolar disorders.

People with depression are diagnosed with psychotic depression if they show positive symptoms simultaneously. J. G. Goekoop and colleagues in the Netherland in a followup study of clinical samples of depression reported that whereas patients with depression as a whole were characterized by higher HA and lower SD than healthy controls during the acute episode and higher HA after full remission, patients with psychotic depression were characterized by lower cooperativeness and lower reward dependence (RD) in the acute episode and lower RD after full remission. Hence it may be that people with psychotic depression share the same personality traits of low RD with people with schizophrenia although the latter may be differentiated by high self-transcendence.

Z. Chen and colleagues in China, in their cross-sectional nonclinical population study, conducted a unique examination of TCI subscale score associations not with the total score of Zung's Self-rating Depression Scale but with the scores of its subscales. Unexpectedly, it was not the negative subscale score but the positive subscale score (consisting of items such as “enjoy things” (reverse) and “feel useful and needed” (reverse)) that was predicted by low SD, cooperativeness, RD, and persistence. This observation shows the importance of the absence of positive emotions in addition to the presence of negative emotions in mood disorders.

Depression is often observed among pregnant women. E. Andriola and colleagues, in Italy, present unique preliminary findings on TCI patterns among expectant mothers and their partners. Both groups were characterized by low SD, whereas only expectant mothers were demonstrated to have high HA.

Eating disorders (ED) are often comorbid with depression, and individuals with both conditions are known to be resistant to treatment. A. D. Giovanni and colleagues, in Italy, report a high prevalence of major depression (MD) in outpatients with ED. Compared to patients with ED only, those with ED and MD demonstrated higher anger and eating disorder pathology scores. They were also characterized by high HA and low SD.

C. R. Cloninger hypothesized dopamine, serotonin, and noradrenaline to be biological substrates of novelty seeking (NS), HA, and RD, respectively. Hence it may be of research interest to investigate the temperaments of patients suffering from conditions characterized primarily by deficiencies of these neurotransmitters. Parkinson's disease (PD) is such an example. PD is known to be caused by dopamine deficiency in cells of the substantia nigra. Pluck and Brown, in the UK, studied PD patients and controls. They found that NS scores correlated with a reaction time measure of attentional orientation to visual novelty, whereas HA scores correlated with anxiety scores. These observations confirm Cloninger's original hypotheses about attention and learning in NS and HA.

Now that we have identified links between temperament and character domain patterns and depression, we must further investigate what mediates these effects. One possible mediator is coping style. M. Fushimi, in Japan, provides a hint that external locus of control is linked to psychological maladaptive patterns. Such coping styles may be based on personality traits. Other promising candidate mediators include self-esteem and self-efficacy, depressogenic dysfunctional attitudes and thinking errors, lack of social supports and social networks, poor coping reaction (rather than perceived coping styles), and stressful life events induced by specific personal traits.

Deeper insight into the association between personality and depression may contribute to the more efficacious treatment of depression.

*Toshinori Kitamura* *Toshinori Kitamura* *C. Robert Cloninger* *C. Robert Cloninger*

